# Case Report: Application of whole exome sequencing for accurate diagnosis of rare syndromes of mineralocorticoid excess

**DOI:** 10.12688/f1000research.8779.2

**Published:** 2017-09-04

**Authors:** Ranjit Narayanan, Shamsudheen Karuthedath Vellarikkal, Rijith Jayarajan, Ankit Verma, Vishal Dixit, Vinod Scaria, Sridhar Sivasubbu

**Affiliations:** 1 Department of Nephrology, KMCT Medical College Hospital, Kerala, India; 2 Academy of Scientific and Innovative Research (AcSIR), CSIR-IGIB South Campus, Delhi, India; 3 Genomics and Molecular Medicine, CSIR Institute of Genomics and Integrative Biology (CSIR-IGIB), Delhi, India; 4 GN Ramachandran Knowledge Center for Genome Informatics, CSIR Institute of Genomics and Integrative Biology (CSIR-IGIB), Delhi, India

**Keywords:** whole exome sequencing, mineralocorticoid excess

## Abstract

Syndromes of mineralocorticoid excess (SME) are closely related clinical manifestations occurring within a specific set of diseases. Overlapping clinical manifestations of such syndromes often create a dilemma in accurate diagnosis, which is crucial for disease surveillance and management especially in rare genetic disorders. Here we demonstrate the use of whole exome sequencing (WES) for accurate diagnosis of rare SME and report that p.R337C variation in the *HSD11B2* gene causes progressive apparent mineralocorticoid excess (AME) syndrome in a South Indian family of Mappila origin.

## Abbreviations

SME: Syndromes of mineralocorticoid excess; AME: Apparent Mineralocorticoid Excess; CAH:17α-hydroxylase: Congenital adrenal hyperplasia due to 17α-hydroxylase deficiency, CAH: 11β-hydroxylase deficiency: Congenital adrenal hyperplasia due to 11β-hydroxylase deficiency; CYP17A1: cytochrome P450, family 17, subfamily A, polypeptide 1; CYP11B1: cytochrome P450, family 11, subfamily B, polypeptide 1, HSD11B2: hydroxysteroid (11β) dehydrogenase 2, *ENaC*; the epithelial sodium channel subunit genes, *WNK1*: WNK lysine deficient protein kinase 1, *WNK4*; WNK lysine deficient protein kinase 1; *KLHL3*: kelch-like family member 3, *CUL3*: cullin 3; *SPAK*: type III secretion system chaperone; *MCR/NR3C2*: Nuclear Receptor Subfamily 3, Group C, Member 2; WES: Whole exome sequencing.

## Introduction

Syndromes of mineralocorticoid excess (SME) are a group of syndromes characterized by an abnormal activation of the amiloride-sensitive sodium channels in the distal tubules of the kidney resulting in an abnormal salt balance. The symptoms are characteristically an abnormality in salt balance (due to an over activation of the channels mediated through the mineralocorticoid receptor), water retention, hypokalemia, low renin levels and hypertension ^1– 3^. The syndrome of apparent mineralocorticoid excess is a rare autosomal recessive form of severe juvenile hypertension which occurs due to mutations in the *HSD11B2* gene ^1^ (OMIM #614232). HSD11B2 is responsible for oxidising glucocorticoid cortisol to inactive cortisone, which does not bind to the mineralocorticoid receptor. Since cortisol binds as avidly as aldosterone to the mineralocorticoid receptor, and the usual plasma cortisol concentration is approximately 100-fold higher than aldosterone, the deficiency of 11β-HSD2 enzyme leads to the persistence of cortisol. This in turn results in marked elevation in net mineralocorticoid activity ^4^. Based on the clinical presentation, differential diagnosis would include Liddle syndrome, Geller syndrome, Gordon syndrome, Apparent Mineralocorticoid Excess (AME) and other milder variants such as CAH:17α-hydroxylase and CAH:11β-hydroxylase deficiency. Mutations in various genes including *CYP17A1, CYP11B1, HSD11B2, ENaC, WNK1, WNK4, KLHL3, CUL3, SPAK* and *MCR/NR3C2* may result in clinical features that resemble SME ^1– 3^. Due to the large diversity and overlapping clinical features, accurate diagnosis is highly reliant on genetic characterization. The need to screen a large number of variants and genes that could cause disease, using conventional approaches such as capillary sequencing is tedious, time consuming and often expensive. Whole exome sequencing (WES) has emerged as an alternative strategy in such clinical settings ^5, 6^.

## Case history

A 30-year-old male, presented to clinic with accelerated hypertension and palpitations. Clinical workup revealed hypokalemia and renal dysfunction. He had a history of hypokalemic paralysis at the age of 15 and was found to be hypertensive at the age of 18. He now had a BP of 170/100 mmHg with no clinical signs of volume overload. There was 1+ albuminuria without any active urinary sediment, while serum creatinine was 2.2 mg/dL and serum potassium was 3 meq/dL. Ultrasound imaging revealed normal sized kidneys with medullary nephrocalcinosis. Blood gas analysis showed a pH of 7.42 with bicarbonate of 24 meq/dL. His 2D echocardiography showed significant left ventricular hypertrophy (LVH). His plasma aldosterone (28 pg/ml) (normal: 30–355 pg/ml) and plasma renin activity (PRA) (0.14 ng/ml/hr) (normal: 1.9–6.0 ng/ml/hr)- were low. He was started on spironolactone (50mg daily) in addition to amlodipine (10mg) and metoprolol (50 mg daily) with which his blood pressure came under control.

His younger brother, who was 28-year-old, had a similar presentation in the form of hypokalemic paralysis at 15 and accelerated hypertension at age 18. The patient’s blood tests from when he was 18 years old, showed serum creatinine levels of 0.7 mg/dL and potassium levels of 2.4 meq/L. Plasma aldosterone (6.5pg/ml) and plasma renin activity (PRA) (0.1 ng/ml/hr) were low on initial evaluation but there was no follow-up for the next few years. When called for evaluation now, his BP was 220/110 mmHg; serum creatinine was 2.0-mg/dL and serum potassium 3.0 meq/L. He did not have any albuminuria. Blood gas analysis showed a normal pH with bicarbonate of 25 mmol/L. He also had medullary nephrocalcinosis on abdominal imaging and significant LVH on 2D echocardiography. He remains extremely irregular with follow-up and still has uncontrolled BP.

**Figure 1.**
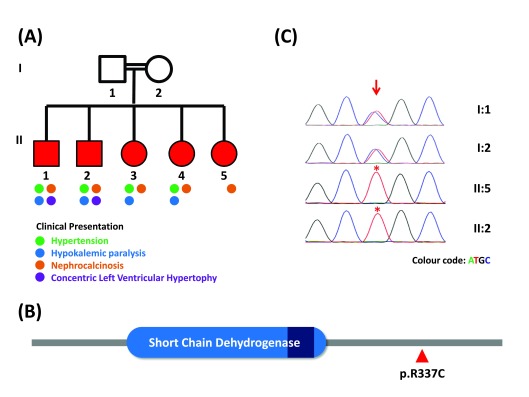
( **A**) Family pedigree marked with progressive phenotypes. ( **B**) Secondary structure of HSD11B2 marked with major domains and the p.R337C amino acid position. ( **C**) Capillary sequencing chromatogram representation of p.R337C variation in the family; arrow and asterisks marks depict the variation loci and affected individuals respectively.

The index patient is the eldest (30 years old) and the brother mentioned (28 years old) is the second of five siblings, the others being females aged 22, 18 and 14 years old ( Figure-1A). The two older sisters have a history of hypertension and hypokalemia. The 18-year-old sister has serum creatinine levels of 1.5 mg/dL, with serum potassium levels of 3.2 meq/L with medullary nephrocalcinosis. While the youngest sibling (14 years old) is normotensive, normokalemic and has normal GFR, her ultrasound results indicated she had medullary nephrocalcinosis. Both parents and their siblings are unaffected by the above syndrome. The parents were first cousins by blood relation. Given the autosomal recessive inheritance pattern and the clinical picture of hypertension, hypokalemia and nephrocalcinosis, a provisional diagnosis of apparent mineralocorticoid excess (AME) was made. The 24-hour urinary cortisol and cortisone estimation was not available.

## Whole exome sequencing, data analysis and validation

Whole blood was collected from the parents and the affected family members after obtaining written informed consent. DNA was extracted from blood and 50 ng of the high quality DNA of the index patient (30 years old) was used to prepare library and exome capture using the Nextera Rapid Capture Expanded Exome kit. The sequencing was performed on Illumina HiSeq 2500 sequencer using v3 reagents (Illumina Inc, USA) to generate over 49.48 million paired end reads of 101bp. The variation calling, annotation and prioritisation were performed as previously described ^6^, which revealed the presence of homozygous C to T transition in the exon 5 (c.1009C>T) of the *HSD11B2* gene (Ensemble transcript ID: NM_000196) causing protein change of p.R337C ( Figure-1B). The variant was found to be pathogenic in ClinVar ^7^ and predicted to be deleterious using PROVEAN ^8^. The variant was further validated using Sanger sequencing of the amplicons, confirming the diagnosis ( Figure-1C). Both the parents were heterozygous for the mutation.

## Discussion

AME is a rare heterogeneous low renin retention SME disorder that manifests with severe juvenile hypertension, hypokalemic alkalosis, low birth weight, failure to thrive, poor growth, and in many cases nephrocalcinosis caused by homozygous and compound heterozygous mutations in the *HSD11B2* gene. The mutations in *HSD11B2* result in a high circulating level of cortisol, and further illegitimate activation of the mineralocorticoid receptor, outcompeting aldosterone and causing activation of the downstream pathways and a clinical presentation of AME symptoms.

Our family presented with features of mineralocorticoid excess, nephrocalcinosis, autosomal recessive inheritance pattern and varying degrees of renal dysfunction with low aldosterone and plasma renin activity (PRA). We used WES to characterize and diagnose this family with apparent mineralocorticoid excess and identified a mutation c.1009C>T (p.R337C) in *HSD11B2*. Human cell line studies have demonstrated that p.R337C mutation leads to the low activity of HSD11B2 due to reduced enzyme stability and causes low-renin hypertension thus resulting in AME ^9^. The p.R337 residue of 11-β-dehydrogenase isozyme 2 enzyme is a recognised mutation site **** and has been reported in Zoroastrians from India and Iran (compound heterozygous for p.R337H and Δp.Y338, age of onset from 8 months), in Persian (p.R337C, age of onset from 4 years) and in Japanese (p.R337H and Δp.Y338, age of onset from 2 years) populations ^10, 11^. The age of onset for the disease manifestation due to p.R337C mutation has been previously described to be as early as 4 years. Unlike previous reports with this mutation where the age of onset has been early (8 months to 4 years), our family did not present with clinical symptoms in early childhood but exhibited or manifested the progressive AME phenotypes with increasing age ( Figure 1A) with the older individuals having more severe clinical manifestations including renal impairment. Patients with identical homozygous mutations from different families have been described to have varying degrees of severity in clinical and biochemical features ^10^. Early diagnosis and prompt institution of salt restriction and spironolactone in these patients could prevent secondary organ damage. Our report also serves to highlight the utility of WES as a tool for diagnosing rare genetic diseases even where biochemical characterization is unavailable.

## Patient consent

Written informed consent for publication of these data were obtained from the patients.

## Data availability

The data referenced by this article are under copyright with the following copyright statement: Copyright: © 2017 Narayanan R et al.

The raw whole exome sequence are available at the NCBI Sequence Read Archive ( http://www.ncbi.nlm.nih.gov/sra), accession number SRR3546815.
