# Supplementary material for: Case Report: Application of whole exome sequencing for accurate diagnosis of rare syndromes of mineralocorticoid excess
Source: F1000Res. 2017 Sep 4;5:1592. Originally published 2016 Jul 6. [Version 2] doi: 10.12688/f1000research.8779.2 (PMC5635450; doi:10.12688/f1000research.8779.2)
Supplement: Peer review history

# Peer review: Referee response for version 2

**DOI:** 10.5256/f1000research.13312.r25633

**Published:** 2017-10-06

**Authors:** Parag Tamhankar

**Affiliations:** 1 Genetic Research Center, National Institute For Research In Reproductive Health, Mumbai, Maharashtra, India

The queries raised previously have been adequately addressed. Hence, I recommend the paper to be accepted in the present form.

I have read this submission. I believe that I have an appropriate level of expertise to confirm that it is of an acceptable scientific standard.

# Peer review: Referee response for version 1

**DOI:** 10.5256/f1000research.9448.r15885

**Published:** 2017-01-09

**Authors:** Shitij Arora

**Affiliations:** 1 Montefiore Medical Center, Albert Einstein College of Medicine, Bronx, NY, USA

A very interesting study. It highlights the increasing use of WES in diagnosing rare diseases. The authors confirm the findings of ES with Sangers and sequence both the parent and the proband. The manuscript needs editing and rearrangement of text. The paragraph on SME and AME needs/can probably be put together as the introduction. Minor grammar corrections.

I have read this submission. I believe that I have an appropriate level of expertise to confirm that it is of an acceptable scientific standard.

# Peer review: Referee response for version 1

**DOI:** 10.5256/f1000research.9448.r16258

**Published:** 2016-10-03

**Authors:** Parag Tamhankar

**Affiliations:** 1 Genetic Research Center, National Institute For Research In Reproductive Health, Mumbai, Maharashtra, India

Please could you add an introduction about syndrome of apparent mineralocorticoid excess, for example;

 Syndrome of apparent mineralocorticoid excess (MIM#218030) is a rare cause of juvenile hypertension occuring due to homozygous or compound heterozygous mutations in HSD11B2 gene (MIM*614232). The gene encodes an NAD+-dependent enzyme viz corticosteroid 11-β-dehydrogenase isozyme 2, which is expressed in aldosterone-selective epithelial tissues such as the kidney, colon, salivary and sweat glands. This enzyme is responsible for oxidizes the glucocorticoid cortisol to the inactive metabolite cortisone. This protective mechanism is necessary because cortisol circulates at 100-1000-fold higher concentrations than aldosterone, and binds with equal affinity to the mineralocorticoid receptor, thereby out-competing aldosterone in cells that do not produce HSD11B2. In patients with SME the enzyme deficiency allows mineralocorticoid receptors to be occupied by cortisol leading to hypertension. 

 I believe that more details on clinical features should be provided, such as:

- clinical presentation and history. Draw three generation pedigree. 
- detailed anthropometry, examination findings of the patients. For eg. was there any low birth weight, short stature, polydipsia, polyuria?
- what were the baseline blood pressure levels in the patients when hypertension was detected?
- was fundus examination done to detect retinopathy?
- were the parents hypertensive?
- give the detailed serum electrolyte levels, detailed 2ECHO report. Give normal ranges of plasma renin, aldosterone levels. 
- clinical photos if relevant
- or uploading ultrasound kidney scans

 

 Mutation: Please give the nucleotide change, exon number of the mutation identified in the patients with the correct Ensembl transcript ID. Please specify as the mechanism of loss of function of the mutation in patients with mutations at position p.335-339 is reduced protein stability due to rapid protein degradation at the proteasome, rather than reduced catalysis.

 Grammar changes: I would begin the words aldosterone, amlodipine, metoprolol with lower case letters when in the midst of a sentence.

 Typo: I think you should correct the following sentence - p.R337 loci of 11HSDB2 to- **“**The p.R337 residue of 11-β-dehydrogenase isozyme 2 enzyme is a recognised mutation site. The mutation p.R337C has been previously identified in a family from Iran with three affected children. **”**

I have read this submission. I believe that I have an appropriate level of expertise to confirm that it is of an acceptable scientific standard, however I have significant reservations, as outlined above.

# Response

**Published:** 2017-07-30

**Authors:** Vinod Scaria

**Affiliations:** Institute of Genomics and Integrative Biology, Delhi, India

Please could you add an introduction about syndrome of apparent mineralocorticoid excess, for example;

Syndrome of apparent mineralocorticoid excess (MIM#218030) is a rare cause of juvenile hypertension occurring due to homozygous or compound heterozygous mutations in HSD11B2 gene (MIM*614232). The gene encodes an NAD+-dependent enzyme viz corticosteroid 11-β-dehydrogenase isozyme 2, which is expressed in aldosterone-selective epithelial tissues such as the kidney, colon, salivary and sweat glands. This enzyme is responsible for oxidizes the glucocorticoid cortisol to the inactive metabolite cortisone. This protective mechanism is necessary because cortisol circulates at 100-1000-fold higher concentrations than aldosterone, and binds with equal affinity to the mineralocorticoid receptor, thereby out-competing aldosterone in cells that do not produce HSD11B2. In patients with SME the enzyme deficiency allows mineralocorticoid receptors to be occupied by cortisol leading to hypertension. 

**We thank reviewers for the comments. Clarifications are as follows:**

Clarification: The suggested changes in introduction section are made.

**1.** **Clinical presentation and history. Draw three generation pedigree.**

Clarification 1: The suggestions have been incorporated. A two generation pedigree is provided. Information about the 3 ^rd^ generation was not available.

**2. detailed anthropometry, examination findings of the patients. For eg. was there any low birth weight, short stature, polydipsia, polyuria?**

Clarification 2: There was no h/o low birth weight, polydipsia or polyuria. All developmental milestones were normally attained in all siblings.

**3. **what were the baseline blood pressure levels in the patients when hypertension was detected?**

Clarification 3: Included in the case history section

**4. was fundus examination done to detect retinopathy?**

Clarification 4: No

**5. were the parents hypertensive?**

Clarification 5: No

**6. give the detailed serum electrolyte levels, detailed 2ECHO report. Give normal ranges of plasma renin, aldosterone levels.**

Clarification 6: Included in the case history section *.*

**7. clinical photos if relevant**

Clarification 7: None

**8. or uploading ultrasound kidney scans**

Clarification 8: **The ultrasound scan is attached separately. It may not be of great quality, however shows medullary nephrocalcinosis (see this link).

**9.** **Mutation: Please give the nucleotide change, exon number of the mutation identified in the patients with the correct Ensembl transcript ID. Please specify as the mechanism of loss of function of the mutation in patients with mutations at position p.335-339 is reduced protein stability due to rapid protein degradation at the proteasome, rather than reduced catalysis.**

Clarification 9: Suggestions have been incorporated.

**10. Grammar changes: I would begin the words aldosterone, amlodipine, metoprolol with lower case letters when in the midst of a sentence.**

Clarification 10: Suggestions have been incorporated.

**11. Typo: I think you should correct the following sentence - p.R337 loci of 11HSDB2 to- “The p.R337 residue of 11-β-dehydrogenase isozyme 2 enzyme is a recognised mutation site. The mutation p.R337C has been previously identified in a family from Iran with three affected children.”**

Clarification 11: Suggestions have been incorporated.
